# The Prognostic Value of Nanog Overexpression in Lung Cancer: A Meta-Analysis

**DOI:** 10.1155/2018/3429261

**Published:** 2018-12-03

**Authors:** Wei Cheng, Hongzhi Wang, Juanjuan Yuan, Ziwei Cheng, Dongwei Xing, Minguang Zhang

**Affiliations:** ^1^Shanghai Municipal Hospital of Traditional Chinese Medicine, Shanghai University of Traditional Chinese Medicine, Shanghai 200071, China; ^2^Cancer Hospital, Chinese Academy of Sciences, Hefei, Hefei, Anhui 230011, China; ^3^Hefei Hospital Affiliated to Anhui Medical University/The Second People's Hospital of Hefei, Hefei, Anhui 230011, China

## Abstract

**Background:**

Recent several studies have showed that the nanog overexpression leads to poor prognosis in some kinds of cancer including hepatocellular carcinoma and gastrointestinal luminal cancer. However, the correlations between prognosis and clinic-pathological features and nanog overexpression in lung cancer are still not well-known. Thus, we performed a meta-analysis to evaluate the role of nanog in lung cancer.

**Methods:**

An electronic retrieval for related studies was conducted in PubMed, Cochrane Library, Web of Science, EMBASE databases, Chinese CNKI, and the Chinese Wan Fang database up to May 2018. The relationships between nanog overexpression and overall survival (OS) and disease-free survival (DFS) as well as clinic-pathological features in lung cancer were investigated. Pooled hazard ratios (HRs) and odds ratios (ORs) with 95% confidence intervals (CIs) were calculated by STATA12.

**Results:**

11 studies containing 1422 patients were identified in our meta-analysis. The overexpression of nanog showed decreased OS (HR = 1.83, 95% CI = 1.49-2.25,* P* ≤ 0.001) and DFS (HR = 1.86, 95% CI = 1.2-2.9,* P* = 0.006). Moreover, overexpression of nanog was significantly related to differentiation (OR = 4.17, 95% CI = 2.17-6.43,* P* ≤ 0.001), lymph node metastasis (OR = 1.76, 95% CI = 1.06-2.91,* P* = 0.028) and tumor size (OR = 1.93, 95% CI = 1.17-3.20,* P *= 0.010), and no correlation with T stage, TNM, stage, and gender.

**Conclusions:**

Our results suggested that nanog overexpression, a hazard factor of differentiation, lymph node metastasis, and tumor size, may predicate decreased OS and DFS for lung cancer.

## 1. Introduction

Lung cancer is ranked at the first in both incidence and mortality worldwide [1, 2]. Significant progress has been made in the diagnosis and treatment of lung cancer in recent years, the etiology of lung cancer is still relatively complicated, and the therapeutic effectiveness is still not satisfactory in Europe and USA [3]. Radical surgery is the main treatment regimen for early lung cancer and these patients have relatively high overall survival (OS) and disease-free survival (DFS). However, most patients with lung cancer are in locally advanced or advanced stage when founded and lose the best time for surgery. Radiotherapy, chemotherapy, and molecular targeted therapy as well as immune therapy are the main treatment for those patients in advanced stage [4, 5]. However, the effectiveness of this treatment is still not very ideal. Therefore, it is very urgent to find an effective prognostic marker and explore new therapeutic targets.

At present, the latest theory shows that tumors are a kind of stem cell disease. Malignant tumors include a small portion of cancer stem cells (CSCs) and act as a pivotal part in the formation and growth of tumors. These CSCs have the ability to multiply, self-renew and differentiate, and express similar molecular markers and gene products [6]. Nanog was reported by Chambers [7] in 2003 as a CSCs marker that plays a decisive role in maintaining cell self-renewal and maintaining pluripotency and also promotes tumor proliferation, invasion, and metastasis. Currently, nanog have been regarded as an indicator of poor prognosis in breast cancer [8], liver cancer [9], gastrointestinal luminal cancer [10], bladder cancer [11], prostate cancer [12], and glioma [13]. Nanog is also reported to be involved in prognostic value and clinic-pathological feature in lung cancer in some studies [14–24]. But the outcomes of the available literatures are inconsistent or even conflicting.

To deeply understanding the relation between nanog overexpression and prognosis in lung cancer, we performed the present meta-analysis to assess the influence of nanog on survival and clinic-pathological parameters in patients.

## 2. Methods and Methods

### 2.1. Search Strategy

The electronic database including PubMed, Cochrane Library, Web of Science, EMBASE database, Chinese CNKI, and the Chinese Wan Fang database was searched (Last update May 2018). Searches contained the terms “nanog or NANOG” (abstract/title) and “cancer or tumor or carcinoma or neoplasm” (abstract/title) and “lung or pulmonary” (abstract/title). All qualified studies were acquired and all references for those selected articles were screened and evaluated. Some review articles were manually retrieved to look for other qualified studies which were then assessed for inclusion by two reviewers (Wei Cheng and Juanjuan yuan). Divergences were resolved by consultation.

### 2.2. Eligibility Criteria

The included criteria for eligible studies in our meta-analysis are (1) patients were diagnosed by histopathologic examinations; (2) the expression of nanog was assayed by immunohistochemistry (IHC) or reverse transcription polymerase chain reaction (RT-PCR); (3) the results contained the survival information (including OS or DFS) or clinic-pathological variables; (4) hazard ratios for OS or DFS can be acquired from studies or calculated from the survival curves.

### 2.3. Data Extraction

The data was extracted from all qualified studies by two authors (Wei Cheng and Juanjuan Yuan). All the extracted data contained first author, publication year, country, number of cases, detect methods of nanog, antibody used, cut-off value of nanog, hazard ratios (HR) and 95% confidence intervals (CIs) (Table 1), and some clinic-pathological variables associated with nanog overexpression, including differentiation, lymph node metastasis, tumor size, T stage, TNM stage, and gender in lung cancer. If data were not available from the primary studies, we contact first author to acquire the information needed.

### 2.4. Quality Assessment

The studies quality was evaluated by Newcastle-Ottawa-Scale (NOS) criteria [25]. The studies with NOS score equal to 6 or higher were considered as high quality; otherwise, they were defined as relative low quality study. All the studies included in our articles were considered to be of good quality.

### 2.5. Statistical Analysis

We divided the original aims into two categories for this meta-analysis. The first aim was to estimate the prognostic value of nanog overexpression on OS and DFS. HRs and 95% CIs were calculated by the survival data extracted from Kaplan-Meier curve with Engauge Digitizer version 4.1 as described before when not directly gained in studies [26–28]. The second interest was to assess the correlation between nanog overexpression and the clinic-pathological variables, including differentiation, lymph node metastasis, tumor size, T stage, TNM stage, and gender. We compared poor and undifferentiation with well and moderate differentiation, lymph node metastasis (YES) with lymph node metastasis (NO), tumor size (≥3 cm) with tumor size (<3 cm), T stage (T3 - T4) with T stage (T1 - T2), TNM stage (III + IV) with TNM stage (I + II), and gender (man) with gender (women). Statistical heterogeneity was estimated with I^2^ test and Q test. We considered heterogeneity present when* P *≤ 0.1 for the Q test or I^2^ > 50%. In the absence of statistical difference for heterogeneity, we used the Mantel-Haenszel method in the fixed-effect model for our meta-analysis. Otherwise, the DerSimonian and Laird method in the random-effect model was chose. The effect of nanog overexpression on survival and clinic-pathological features was evaluated with pooled HRs and ORs with 95% CIs. Publication bias was assessed by Begg's funnel plot and Egger's test [29, 30].* P* < 0.05 was considered as statistical difference [31]. The source of heterogeneity was detected by subgroup analysis and sensitivity analysis. Statistical analyses were conducted with STATA version 12.0.

## 3. Results

### 3.1. Study Characteristics and Quality Assessment

**Figure 1:**
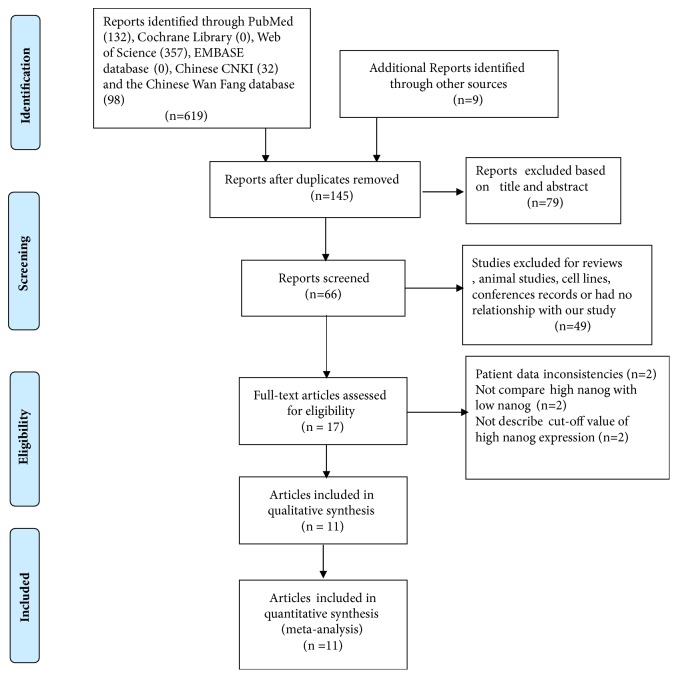
Flow diagram of the study selection in this meta-analysis.

619 potential relevant studies were searched from the databases according to the search terms. As shown in Figure 1, duplicated studies, conferences records, animal studies, and some nonoriginal articles (such as review, letter) were eliminated by checking the titles and abstracts (602). The remaining 17 articles were further screened by reading full text. Another 6 articles were eliminated due to patient data inconsistencies (n=2), not including comparison between high nanog and low nanog (n=2) and no description about cut-off value of high nanog expression (n=2). Finally, 11 eligible [14–24] studies with 1422 lung cancer patients were included. The publication time were from 2010 to 2017 and the sample size from 50 to 309 patients (Table 1). 7 studies were from China; 7 studies were published in English, and the others were in Chinese. All studies were of good quality with NOS scores ≥ 6.

### 3.2. Association between Nanog Overexpression and OS and DFS for Lung Cancer

**Figure 2:**
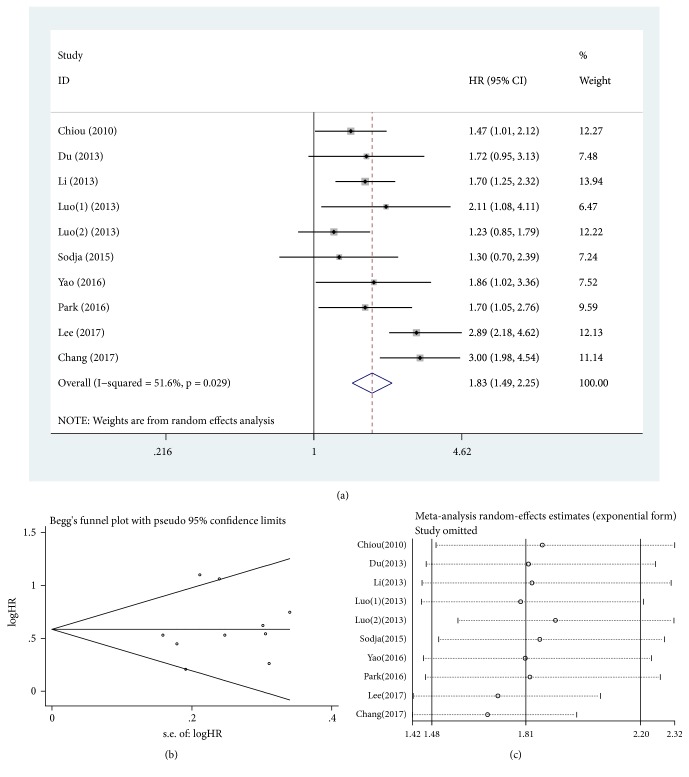
Pooled analysis for the association between nanog overexpression and OS. (a) Forest plots. (b) Funnel plots. (c) Sensitive analysis. OS, overall survival. HR, hazard ratio; CI, confidence intervals; se, standard error.

**Figure 3:**
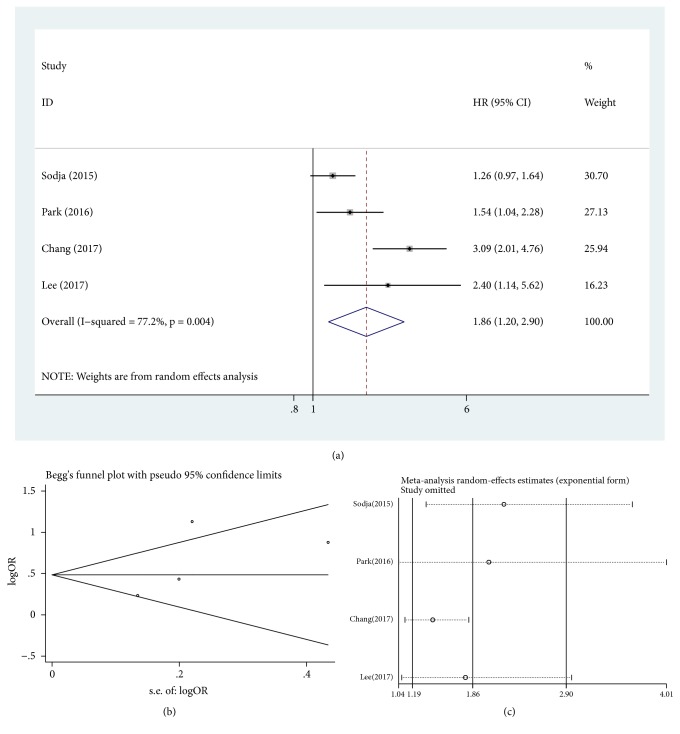
Pooled analysis for the association between nanog overexpression and DFS. (a) Forest plots. (b) Funnel plots. (c) Sensitive analysis. DFS, disease-free survival; HR, hazard ratio; CI, confidence intervals; se, standard error.

Heterogeneity was significant among the included studies when assessing relationship between nanog overexpression and OS and DFS for lung cancer (*P* = 0.029, I^2^ = 51.6%, Figure 2(a), and* P* = 0.004, I^2^ = 77.2%, Figure 3(a), respectively). Meta-analysis with random-model indicated that lung cancer patients with nanog overexpression had significantly decreased OS and DFS (HR = 1.83, 95% CI = 1.49-2.25,* P* ≤ 0.001, Figure 2(a), and HR = 1.86, 95% CI = 1.20-2.90,* P *= 0.006, Figure 3(a), respectively). Lung cancer patients with high nanog expression contributed to shorter OS and DFS when compared with low nanog expression.

### 3.3. Relationship between Nanog Overexpression and Clinic-Pathological Features for Lung Cancer

**Figure 4:**
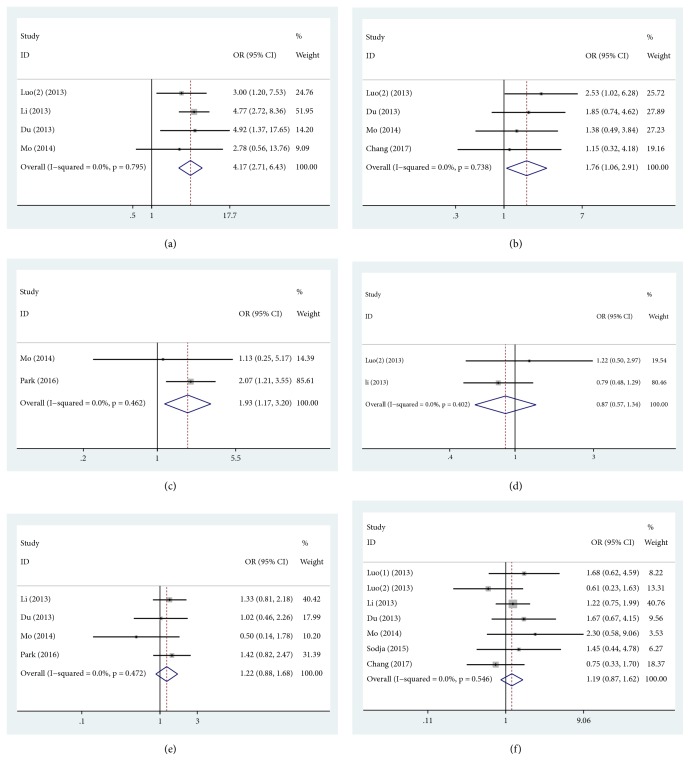
Pooled analysis for the association between nanog overexpression and clinic-pathological features. (a) Differentiation. (b) Lymph node metastasis. (c) Tumor size. (d) T stage. (e) TNM stage. (f) Gender. OR, odds ratio; CI, confidence interval.

The relationship between nanog overexpression and clinical variable in lung cancer was estimated in our meta-analysis (Table 2). Nanog overexpression was significantly related to differentiation (poor and undifferentiation versus well and moderate differentiation: OR = 4.17, 95% CI = 2.71-6.43,* P* ≤ 0.001, fixed effect, Figure 4(a)), lymph node metastasis (YES versus NO: OR = 1.76, 95% CI = 1.06-2.91,* P* = 0.028, fixed effect, Figure 4(b)), and tumor size (≥3cm versus < 3cm: OR = 1.93, 95% CI = 1.17-3.20,* P* = 0.010, fixed effect, Figure 4(c)). However, nanog overexpression was not correlated with T stage (T3 - T4 versus T1 - T2: OR = 0.85, 95% CI = 0.57-1.34,* P* = 0.541, fixed effect, Figure 4(d)), TNM stage (III + IV versus I + II: OR = 1.22, 95% CI = 0.88-1.68,* P* = 0.227, fixed effect, Figure 4(e)), and gender (man versus women: OR = 1.19, 95% CI = 0.87-1.62,* P* = 0.287, fixed effect, Figure 4(f)). These results revealed that nanog overexpression conferred poor differentiation and undifferentiation, lymph node metastasis, and tumor size (≥ 3cm) and no influence with T stage (T3-T4), TNM stage (III + IV), and gender.

### 3.4. Subgroup Analysis

Subgroup analysis was used to explore possible sources of heterogeneity among OS (Table 3) and DFS (Table 4) based on pathological types, publication year, NOS score, and country. Ultimately, publication year rather than the pathological types, NOS score, and country might be considered as a sources of heterogeneity for OS and DFS. Our results showed that there was no significant heterogeneity in both subgroups by publication year for OS and DFS (Tables 3 and 4). Nanog overexpression was significantly correlated with poor OS (publication year 2010-2016 subgroup: HR = 1.56, 95% CI = 1.33-1.83,* P *** ≤ **0.001 and publication year 2017 subgroup: HR =2.94, 95% CI = 2.22-3.88,* P* ≤ 0.001; Table 3) and poor DFS (publication year 2010-2016 subgroup: HR = 1.34, 95% CI = 1.08-1.67,* P* = 0.008 and publication year 2017 subgroup: HR = 2.92, 95% CI = 2.00-4.26,* P* ≤ 0.001; Table 4) in lung cancer. Although the pathological types were not the main source of heterogeneity, there was not significant heterogeneity found for OS and DFS in adenocarcinoma subgroup (*P* = 0.375, I^2^ = 5.6%, and* P* = 0.189, I^2^ = 41.9%, respectively). Our outcome also indicated that high nanog expression was associated with poor OS and DFS compared with low nanog expression in adenocarcinoma subgroup (HR = 1.68, 95% CI = 1.34-2.11,* P* ≤ 0.001, and HR = 1.85, 95% CI = 1.16-2.96,* P *= 0.010, respectively). Finally, our subgroup analysis showed that nanog overexpression was always linked to shorter OS and DFS in lung cancer.

### 3.5. Publication Bias

Begg's funnel plot and Egger's test were performed to evaluate the publication bias for OS and DFS in lung cancer patients in included studies. As shown in Figures 2(b) and 3(b), there was no obvious publication bias for OS (Begg's test of* P *= 0.655 and Egger's test of* P* = 0.656; Figure 2(b)) and DFS (Begg's test of P = 0.308 and Egger's test of P = 0.342; Figure 3(b)).

### 3.6. Sensitive Analysis

In order to appraise the effect of single study on the pooled HRs in OS and DFS because of significant heterogeneity, we carried out sensitivity analysis by estimating the average HRs in the absence of each study. The results demonstrated that our meta-analysis was statistically reliable (Figures 2(c) and 3(c)).

## 4. Discussion

Lung cancer is a malignant tumor with high morbidity and mortality. Although there are many treatment strategies for lung cancer, the therapeutic effectiveness is still not satisfactory. It is emergency for us to explore the new mechanism of metastasis and recurrence and look for related prognostic markers and targets of therapeutic interventions to improve the prognosis for lung cancer.

More and more studies have showed that there is a small count of cells with self-renewal and differentiation in tumors. Their characteristics are similar to normal stem cells. We call them CSCs. Increased CSCs are often associated with tumor progression, relapse, and drug resistance [32, 33]. The CSCs related surface markers mainly contain CD133, CD44, and EpCAM, as well as CD90 [34]. Nanog was also considered to be surface marker of CSCs and targets of anticancer therapy in lung cancer in recent studies [35, 36]. So therapy of targeting nanog was also a very promising treatment strategy in lung cancer. Some studies have indicated that nanog was correlated with prognostic value and clinic-pathological features in lung cancer in recently years. But their outcomes are still inconsistent. So we carried out a meta-analysis to estimate the prognostic value of nanog on OS and DFS, as well as the clinic-pathological features in lung cancer.

Our outcomes showed that nanog overexpression have been involved with poor OS and DFS in lung cancer. For clinic-pathological features involved in lung cancer, we found that nanog overexpression was associated with differentiation, lymph node metastasis, and tumor size. The reasons for this may be that nanog can promote invasion, metastasis, and cell proliferation in lung cancer. Our results also demonstrated that no obvious relation was found between nanog overexpression and T stage, TNM stage, and gender. Because of obvious heterogeneity in OS and DFS, we carried out subgroup analysis based on pathological types, publication year, NOS score, and country. Our subgroup results showed that publication year might be considered as a source of heterogeneity. There was no significant heterogeneity in both publication year subgroup (2010-2016) and publication year subgroup (2017) for OS and DFS. Our results indicated that nanog overexpression led to poor OS and DFS in two subgroups. Du to absence heterogeneity for OS and DFS in adenocarcinoma subgroup based on pathological types, our outcome also demonstrated that there was a decreased OS and DFS for lung adenocarcinoma patients with high nanog expression compared with low nanog expression. We performed sensitivity analyses by evaluating the average HRs in the absence of each study. The results indicated that our meta-analysis was statistically reliable. And currently, the heterogeneity cannot be well elaborated and still requires some high quality studies with large sample. In short, our results suggested that nanog overexpression may indicate poor OS and DFS and susceptibility to poor differentiation and undifferentiation, lymph node metastasis, and tumor size (≥3cm). So, it is possible for us to improve OS and DFS for patients with lung cancer by targeting nanog therapy in future. We can also consider determining treatment strategies according to nanog expression level.

Publication bias is an important limitation in meta-analysis, because some studies with negative results are more difficult to be accepted for publication. Thus we should encourage some researchers to publish their studies including some negative results. Our results demonstrated that no significant evidence of publication bias was found in our included studies.

There are still so many other limitations in our study. First of all, prognosis data extracted from survival curves might be less reliable than reported directly in studies. Second, the antibody used, IHC cell-scoring method, and the cut-off value were not defined similarly in partial studies. Third, the heterogeneity of the OS and DFS is significant, although we performed subgroup analysis and sensitivity analyses. These factors may contribute to potential publication bias. Our sensitivity analyses indicated that the results were stable and the heterogeneity did not influence the analysis results.

To summarize, our results demonstrated that nanog overexpression, a hazard factor of differentiation, lymph node metastasis, and tumor size, may contribute to poor OS and DFS for lung cancer. Nanog might be a bad prognostic marker for lung cancer.

## Figures and Tables

**Table 1:** Characteristics of studies included in the meta-analysis.

First author -year	Patient source	Study design	Number of patients Total (P/N)	Method	Antibody	Cut-off	Follow-up (months)	HR estimation	HR (95% CI) (OS)	Survival	Study quality (NOS)
Chiou-2010	China	R	118 (78/40)	IHC	CST	Position	80	Sur-curve	1.47 (1.01-2.12)	OS	7
Li-2013	China	R	309 (94/215)	IHC	CST	5%	69.5	HR	1.70 (1.25-2.32)	OS	8
Du-2013	China	R	123 (98/25)	IHC	CST	Score 8	60	Sur-curve	5.48 (1.42-21.12)	OS	7
Luo (1)-2013	China	R	62 (30/32)	IHC	CST	Position	60	HR	2.11 (1.08-4.11)	OS	6
Luo (2)-2013	China	R	106 (26/80)	IHC	CST	25%	60	Sur-curve	1.23 (0.85-1.79)	OS	6
Mo-2014	China	R	50 (36/14)	IHC	CST	Position	No data	No data	No data	No data	6
Sodja -2015	Slovenia	R	50 (25/25)	RT-PCR		median	32.5	HR	1.30 (0.70-2.39)	OS, DFS	6
Park-2016	Korea	R	226 (96/130)	IHC	Epitomics	Grade 2	125	HR	1.70 (1.05–2.76)	OS, DFS	8
Yao-2016	China	R	156 (129/27)	IHC	Proteintech	10%	25	HR	1.86 (1.02-3.36)	OS	6
Chang-2017	Korea	R	112 (44/68)	IHC	CST	Position	65	HR	3.00 (1.98–4.54)	OS, DFS	8
Lee-2017	Korea	R	110 (55/55)	IHC	CST	Position	65	HR	2.89 (2.18-4.62)	OS, DFS	7

R, retrospective; P, positive; N, negative; IHC, immunohistochemistry; HR, hazard ratios; CI, confidence interval; OS, overall survival; DFS, disease free survival; NOS, Newcastle-Ottawa-Scale.

**Table 2:** The associations between nanog overexpression and clinic-pathological features for lung cancer.

Heterogeneity							

Clinic-pathological features	No. of studies	No. of patients	Pooled OR (95% CI)	PHet	I^2^	P value	Model used

differentiation	4	588	4.17 (2.71-6.43)	0.795	0.0%	≤0.001	Fixed
lymph node metastasis	4	391	1.76 (1.06-2.91)	0.738	0.0%	0.028	Fixed
tumor size	2	276	1.93 (1.17-3.20)	0.462	0.0%	0.010	Fixed
T stage	2	432	0.85 (0.57-1.34)	0.402	0.0%	0.541	Fixed
TNM	4	708	1.22 (0.88-1.68)	0.472	0.0%	0.227	Fixed
gender	7	812	1.19 (0.87-1.62)	0.546	0.0%	0.287	Fixed

Random, random-effects model; fixed, fixed-effects model; OR, odds ratio; CI, confidence interval; NO, number of sample size.

**Table 3:** Subgroup analysis of OS by pathological types, publication year, NOS score, and country.

Subgroup	No. of studies	No. of patients	P value	Pooled HR (95% CI)	PHet	I^2^ (%)
Pathological types						
Adenocarcinoma	5	613	≤0.001	1.68 (1.34-2.11)	0.375	5.6%
Squamous cell carcinoma	2	105	0.270	1.97 (0.59-6.55)	0.009	85.4%
Small cell carcinoma	1	50	0.402	1.74 (0.70-2.23)		
Publication year						
2010-2016	8	1150	≤0.001	1.56 (1.33-1.83)	0.819	0.0%
2017	2	222	≤0.001	2.94 (2.22-3.88)	0.896	0.0%
NOS score						
<7	4	374	0.004	1.46 (1.12-1.89)	0.432	0.0%
≥7	6	998	≤0.001	2.01 (1.55-2.60)	0.038	57.6%
Country						
China	6	874	≤0.001	1.57 (1.32-1.87)	0.671	0.0%
Other	4	498	≤0.001	2.20 (1.52-3.19)	0.051	61.5%

OS, overall survival; NO, number of sample size; HR, hazard ratio; CI, confidence interval; NOS, Newcastle-Ottawa-Scale.

**Table 4:** Subgroup analysis of DFS by pathological type, publication year, NOS score, and country.

Subgroup	No. of studies	No. of patients	P value	Pooled HR (95% CI)	PHet	I^2^ (%)
Pathological type						
Adenocarcinoma	2	290	0.010	1.85 (1.16-2.96)	0.189	41.9%
Squamous cell carcinoma	1	48	≤0.001	3.76 (1.89-7.49)		
Small cell carcinoma	1	50	0.085	1.26 (0.97-1.64)		
Publication year						
2010-2016	2	276	0.008	1.34 (1.08-1.67)	0.402	0.0%
2017	2	222	≤0.001	2.92 (2.00-4.26)	0.585	0.0%
NOS score						
≤7	1	50	0.085	1.26 (0.97-1.64)		
>7	3	448	0.001	2.21 (1.36-3.61)	0.061	64.2%
Country						
China	0	0				
Other	4	498	0. 006	1.86 (1.20-2.90)	0.004	77.2%

DFS, disease free survival; NO, number of sample size; NOS, Newcastle-Ottawa-Scale; HR, hazard ratio; CI, confidence interval.

## Abbreviations

OS: Overall survival
DFS: Disease-free survival
HRs: Hazard ratios
ORs: Odds ratios
CIs: Confidence intervals
CSCs: Cancer stem cells
IHC: Immunohistochemistry
RT-PCR: Reverse transcription polymerase chain reaction
NOS: Newcastle-Ottawa-Scale.
